# Retinal Glaucoma Public Datasets: What Do We Have and What Is Missing?

**DOI:** 10.3390/jcm11133850

**Published:** 2022-07-02

**Authors:** José Camara, Roberto Rezende, Ivan Miguel Pires, António Cunha

**Affiliations:** 1 Departamento de Ciências e Tecnologia, Universidade Aberta, 1250-100 Lisboa, Portugal; jcrcamara@hotmail.com (J.C.); al72763@alunos.utad.pt (R.R.); 2 Escola de Ciências e Tecnologia, University of Trás-os-Montes e Alto Douro, Quinta de Prados, 5001-801 Vila Real, Portugal; impires@it.ubi.pt; 3 Instituto de Telecomunicações, Universidade da Beira Interior, 6200-001 Covilhã, Portugal; 4 Instituto de Engenharia de Sistemas e Computadores, Tecnologia e Ciência, 3200-465 Porto, Portugal

**Keywords:** glaucoma, retinal images, databases, glaucoma screening, machine learning

## Abstract

Public databases for glaucoma studies contain color images of the retina, emphasizing the optic papilla. These databases are intended for research and standardized automated methodologies such as those using deep learning techniques. These techniques are used to solve complex problems in medical imaging, particularly in the automated screening of glaucomatous disease. The development of deep learning techniques has demonstrated potential for implementing protocols for large-scale glaucoma screening in the population, eliminating possible diagnostic doubts among specialists, and benefiting early treatment to delay the onset of blindness. However, the images are obtained by different cameras, in distinct locations, and from various population groups and are centered on multiple parts of the retina. We can also cite the small number of data, the lack of segmentation of the optic papillae, and the excavation. This work is intended to offer contributions to the structure and presentation of public databases used in the automated screening of glaucomatous papillae, adding relevant information from a medical point of view. The gold standard public databases present images with segmentations of the disc and cupping made by experts and division between training and test groups, serving as a reference for use in deep learning architectures. However, the data offered are not interchangeable. The quality and presentation of images are heterogeneous. Moreover, the databases use different criteria for binary classification with and without glaucoma, do not offer simultaneous pictures of the two eyes, and do not contain elements for early diagnosis.

## 1. Introduction

Glaucoma is an optic neuropathy of asymptomatic progression and characteristic visual field loss that can lead to total and irreversible blindness. It is estimated that in 2013, 64.3 million people aged 40 to 80 years were diagnosed with glaucoma, and this number is expected to increase to 76 million by 2020 and 111.8 million by 2040 [1]. In most cases, the disease has a slow and asymptomatic evolution. At the time of diagnosis, many patients have already had some degree of visual damage with varying degrees of disability for work and activities of daily living. The situation worsens with a lack of specialists and equipment, and many cases may be under-reported. Therefore, there is a need to popularize glaucoma screening through cheaper techniques that serve many people. Early diagnosis and treatment can delay the progression of glaucomatous disease. Currently, it is estimated that half of all glaucoma patients remain undiagnosed. Detecting this substantial number of undiagnosed patients is a significant challenge [2].

In recent years, deep learning techniques have demonstrated the potential to solve complex problems involving images and medical domains, such as automated glaucoma screening. For example, in visual field examination, deep learning has been shown to differentiate between normal visual fields and those with pre-perimetric glaucoma [3]. Still, the scope of glaucoma screening using these techniques is not yet defined. 

In medicine, the use of artificial intelligence (AI), through deep learning techniques, has shown potential for population screening and reducing glaucoma progression in patients waiting for help from professionals, facilitating populations’ access to early diagnoses, especially in more distant locations, which standardizes diagnostic results and even decreases diagnostic divergence between specialists. However, AI offers only one reference for a clinical diagnosis. Thus, medical specialists will always be responsible for diagnosis and treatment [4]. In other medical areas, deep learning methodologies are used to segment lungs, brain, cell mitosis, prediction of development, progression of myopia [4], and diagnostics in the digestive system. So far, in ophthalmology, two AI algorithms have been approved by the FDA for clinical use. One is IDX-DR, which can detect diabetic retinopathy (DR), and the other is Viz.AI, which analyzes images for indicators associated with a stroke [4].

Color fundus photography (CFP) is a cost-effective and non-invasive way to analyze the retina [5,6]. CFPs are considered a significant element in glaucoma screening in clinical practice and deep learning methodologies. Public databases contain a set of color photographs used for training deep learning (DL) algorithms intended to screen for various retinal pathologies such as diabetic retinopathy, diabetic macular edema, and glaucoma. In clinical practice, CFPs are analyzed with other clinical data such as intraocular pressure measurements (IOP), automated perimetry, and optical coherence tomography (OCT) to diagnose glaucoma. However, large-scale population screening programs based on traditional clinical methodologies are not employed, as they are uneconomic [7]. In addition, they can generate many false positives, creating a burden on public health infrastructure and a harmful condition on the patient’s quality of life until the glaucoma diagnosis is ruled out [8]. 

Public databases have diverse characteristics that make it difficult to correlate data. For example, some databases present the optic disc (OD) segmentation and cupping made by experts and grouped into sets of “normal” and “glaucomatous” papillae based on appearance. Others give classifications based on patient chart data and show evolving stages of glaucoma, i.e., an early, moderate, and severe glaucoma group.

Other retinal pathologies concomitant with glaucoma should be evaluated in clinical practice. The specialist can diagnose them through clinical data or examination findings. Color retinography is an element of immense importance for diagnostic aid and will undoubtedly demand an excellent function of the algorithms to embrace a more comprehensive diagnostic range. 

In the development of deep learning databases and architectures, a significant research effort is being made to introduce tools for the segmentation of the OD and the optic cup in CFPs and to identify glaucoma cases based on clinical features [9,10,11]. However, the approaches used in different databases cannot be adequately compared due to the lack of a single validation protocol [8] and the limited size of the available datasets. In addition, the absence of large-scale glaucoma imaging datasets has hampered the rapid deployment of DL techniques to detect the pathology [12] and the lack of a strategy for marking papillae and excavation boundaries (BENCHMARK) and hinders uniform comparison of existing methods [8].

The optic papilla region includes several anatomical variations considered normal in the population. In addition to spatial and non-overlapping differences between the images of right and left papillae, these variations include the insertion of the nerve in the eye, the size, and excavation of the papilla, vascular emergence, pigmentation, and the peripapillary region. Many variables may be presented as different fingerprints in a population capable of influencing the training and the results of neural networks. Therefore, there is a requirement to constantly update the datasets and establish protocols for the images integrated with clinical data and ancillary tests. In addition, there is a need to define the training and validation set of the public databases, develop automated tools for segmentation and classification of the CFP to reduce data deficiency, and ensure more reliable results for effective glaucoma detection. Public databases adapt to these requirements through the challenges for segmentation and classification as in REFUGE and, more recently, another challenge, “Artificial Intelligence for RObust Glaucoma Screening” (AIROGS). 

This study aims to analyze the gold standard public databases for glaucoma analysis and offer recommendations to improve their contributions to the learning of neural networks showing significant advances for automated screening of glaucomatous papillae. It is divided into five main topics: a few fundamentals of glaucoma, deep learning in the context of artificial intelligence, fundamentals of the existing public databases for the study of the retina, and fundamentals considered the gold standard for the study of glaucoma. Finally, various recommendations will be analyzed to improve existing databases and those used in the future.

## 2. Fundamentals of Glaucoma

Some fundamentals of glaucoma will be analyzed with an emphasis on clinical diagnosis. Specifically, classification using fundus photographs, the most used ancillary tests, and the principles used in the treatment will be discussed.

### 2.1. Clinical Definition and Diagnosis

**Figure 1:**
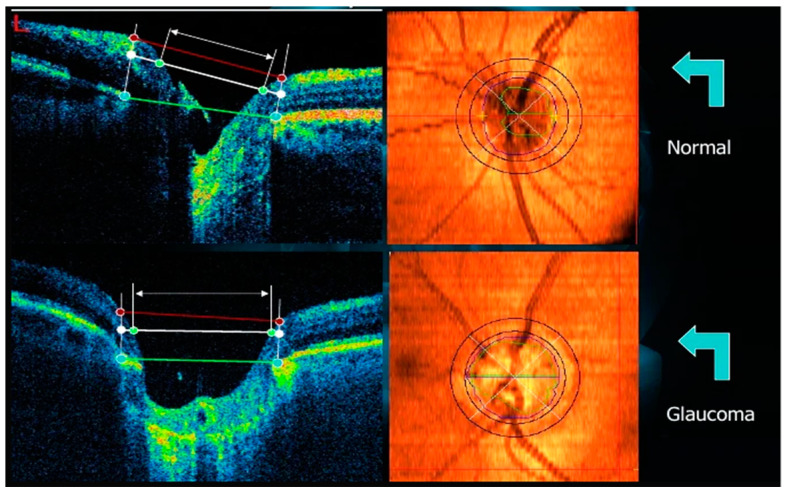
Example of an OCT scan.

Glaucoma is a chronic optic neuropathy characterized by irreversible damage to the retinal nerve fiber layer (RNFL) and characteristic visual field changes. Changes in the nerve fiber layer can be visually translated by irregularities in the inner border of the neuroretinal layer (Figure 1) and increased excavation of the papillae either symmetrically or asymmetrically between eyes. 

Screening for glaucoma should be performed routinely and includes detailed medical history, slit lamp examination, CFPs, and tonometry. CFPs are essential in screening and studying disease progression and are used to study the optic nerve structure in glaucoma. However, identifying glaucomatous features in the optic papilla is challenging even for specialists and requires expertise and years of practice. Some of the main glaucoma-related changes are visualized in the neural layer of the optic papilla, including increased cupping and neuroretinal thinning, as well as other changes earlier in the peripapillary region, such as retinal fiber layer defects and peripapillary atrophy [13]. In addition, the disease may progress asymmetrically between the two eyes, with a normal eye (without cupping) and a counter lateral eye with a larger cup or even a cup of assorted sizes between the eyes. 

The most common types of glaucoma have a primary cause, i.e., they originate from ocular factors (not always determined). They can be grouped according to the angle of outflow of the aqueous humor into open-angle glaucoma and closed-angle glaucoma [14]. There is no specific anatomical cause in open-angle glaucoma, although the intra-ocular pressure represents the leading risk factor when elevated. Other risk factors include myopia, increasing age, the Black race, history of the disease in first-degree relatives, vasculopathy, hypertension, and diabetes. Closed-angle glaucoma is associated with anatomical blockage of the aqueous humor drainage angle in the anterior chamber resulting in increased ocular pressure.

In current clinical practice, CFP features are used in conjunction with other clinical data, such as IOP, automated perimetry, and the use of OCT to diagnose glaucoma. However, these approaches are not cost-effective for large-scale population screening for glaucoma [7]. 

Subsidiary tests may be indicated in suspicious cases and the study of glaucoma progression, especially in OCT and the visual field.

#### 2.1.1. Subsidiary Examinations

The early detection of glaucoma includes structural and functional tests. Structural tests aim to detect structural changes in the retinal fiber layer. Structural tests include confocal laser ophthalmoscopy, OCT, and laser polarimetry. Functional tests such as frequency doubling perimetry and short-wavelength automated perimetry, also known as blue-on-yellow perimetry, are intended to detect visual field damage before changes in visual field examination. These tests have limitations, including the inability to assess eyes with high myopia, large ODs, and areas of peripapillary atrophy [15]. In these cases, we must evaluate the patient as a whole and correlate the ocular pressure and structural and functional data.

#### 2.1.2. Visual Field 

Visual field examination, campimetry, and automated achromatic perimetry (white-on-white) have significant importance in diagnosing and evolving glaucoma. Graphic alteration locates the non-vision region corresponding to the neural layer damage by glaucomatous neuropathy. This scan evaluates 30 degrees where most glaucomatous defects occur and can show changes only in the stages where considerable retinal fiber loss has already occurred [16,17], limiting its use in the evaluation of the early stages of the disease. The characteristic visual field alterations correspond to focal or arcuate visual losses in the projection path of the fibers that run through the retina towards the optic papilla and increase with the disease’s progression.

#### 2.1.3. Optical Coherence Tomography 

Optical coherence tomography is a diagnostic aid exam in several retinal pathologies. It shows sections of the retinal layers. In glaucoma, it is used in suspected cases with structural changes confirmed by fundus photography (retinography) when the cup-to-disc ratio ≥ 0.5 and <0.9, in the presence of asymmetry between the two eyes ≥ 0.2 and localized thinning of the neural ring, or for diagnostic clarification in ocular hypertensive patients (ocular pressure above 21 mmHg). It makes it possible to diagnose and monitor glaucoma through the evolution of macular thickness. Although central vision is often preserved in late glaucoma, the thinning of the macular area is a parameter found in the early stages of the disease and precedes visual field defects [18]. Figure 1 exemplifies an OCT diagram (left part) correlating with a papilla (right part). The upper papilla has a standard feature, and the lower papilla has a glaucomatous feature (increased cupping and vessel deflection). Note that the red dots correspond to the size of the papilla, and the blue dots correspond to the size of the cupping, and the blue and yellow dots correspond to the layer of ganglion fibers, which are visibly more minor in the lower diagram.

### 2.2. Principles Used in the Treatment of Glaucoma

Once the diagnosis is established, treatment should be started as early as possible. The treatment of glaucoma aims to stop the progression of the disease. It is achieved using traditional surgical strategies and laser application to increase intraocular fluid filtration and topical or systemic pharmacological approaches to reduce the target eye pressure and stop disease progression. In addition to these strategies, systemic factors such as diabetes, hypertension, tumors, rheumatic processes, and ocular factors, such as cataracts, vasculopathy, malformations, post-traumatic injuries, infections, and hemorrhages should be stabilized.

## 3. Fundamentals of Deep Learning in the Context of AI

Some fundamentals of deep learning within artificial intelligence will be analyzed, with an emphasis on application, the challenges of large-scale application, and ways of applying DL architectures in automated glaucoma classification.

**Figure 2:**
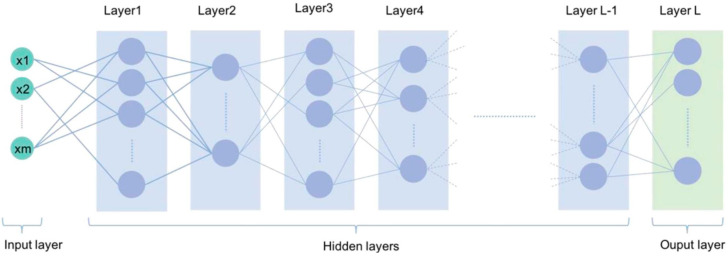
Illustration of a typical deep learning network with multiple layers between the input and output levels (acquired from [4]).

As a subarea of artificial intelligence, machine learning (ML) algorithms deal with large datasets such as thousands of images, facilitating the resolution of problems that would be impractical through classical statistical analysis. In turn, deep learning is a sub-area of machine learning that operates data analysis through the representation of successive layers (neural networks) inspired by the human brain. Each layer could filter specific properties and select more relevant characteristics that have significant applications in medical diagnosis problems, allowing complex representations to be learned and divided into intermediate spaces (layers). Deep learning has demonstrated a vast applicability potential in the medical area by improving image accuracy, processing, and identification of diagnostic relevant features in radiographic images, tomography, ultrasonography, histological analysis of organs and tissues, and photographic analysis images. Deep learning can identify features within a complex structure in large datasets using multiple intermediate layers positioned between the input and output layers (as seen in Figure 2), allowing each layer to learn to transform its input signal to the next layer. It has shown considerable utility in discovering intricate structures in complex data such as medical images. The key to the successful operation of these methods is having enough data to train and evaluate the system. Furthermore, the validation of these methods requires a reference standard that can be used for comparison, i.e., having public retinography databases that satisfy several requirements, which should also be clearly defined [19].

The deep learning workflow can be defined in three steps: (1) pre-processing of image data; (2) model training, model validation, and testing; and (3) evaluation. Pre-processing includes noise reduction, feature selection and extraction from the image, and data normalization. A model to be trained is initially divided into three sets: training data, validation, and testing. The training set allows the model to learn to fit the data parameters of the classifier. The validation set prevents overfitting. The test set is used to evaluate the performance of the trained model. The data provided by the public databases will be used in the training step of the deep learning algorithms. For best results, the data need to be dependable and offered in a sufficient quantity to train and evaluate the system.

### 3.1. Challenges in Applying Deep Learning on a Large Scale

Many of the main challenges for problem-solving through DL architectures are generated by protocol deficiencies in defining public databases. If the databases do not appropriately train the networks, it may be misleading to networks’ responses. Gold standard public databases such as DRIHTI-GS, RIM-ONE DL, and REFUGE offer a reduced dataset and few data for training/testing. For example, data are classified into glaucoma and non-glaucoma groups based on imaging alone in the case of the RIM-ONE DL database, and there is no information on which clinical bases the clinical classification used in the DRISHT-GS1 and REFUGE databases were based. Furthermore, no public database presents images of the papillae of both eyes. The lack of standardized strategies makes it difficult to compare existing methods [8].

According to [4], automated DL techniques may provide false negative results from ocular diseases such as optic neuropathy coexisting with pathological myopia, retinal detachment, and senile macular degeneration. False-positive results arise from other ocular conditions, including increased physiological cupping. In addition, other challenges affect the accuracy of diagnostic results and represent obstacles in large-scale applications of AI technology, such as few standardized sets for training, a limited number of datasets with low anatomical representativeness of normal and glaucomatous papillae, and differences in the quality of images used in different countries, regions, and medical institutions. This inevitably affects the accuracy of image analysis, which may represent a higher computational expense and produce inaccurate results, especially in the early stages of the disease that require the analysis of peripapillary fibers. Furthermore, AI cannot provide the physician or other users with the rationale for the diagnosis. There is no explanation for why the differences exist or the pathological basis of the differences that could affect physicians’ acceptance of these devices in clinical applications. The bases do not show images of the right and left eyes of the same patient, an element widely used in clinical practice for the comparison of cup size because of the asymmetric feature in disease progression. 

### 3.2. Automated Classification of Glaucoma

Automated recognition of glaucomatous papillae in CFPs by DL techniques can be performed in two ways: by directly recognizing glaucomatous features in the optic papilla by DL architectures and by segmenting the disc and excavation of the optic papilla. Existing DL approaches are based on adaptations of supervised DL techniques [8], i.e., techniques capable of “automatically learning” features by analyzing large training sets of segmented images [20] not offered by databases. Through deep learning classifiers, multiple retinal vascular diseases may be distinguished with an accuracy above 80% and may be a valuable instrument in areas with a shortage of ophthalmic care [21].

#### 3.2.1. Classification of Glaucoma Directly through Deep Learning Architectures

Automated glaucoma classification directly by DL architectures classifies an input image as glaucoma or non-glaucoma based on the visual characteristics of the optic papilla and the segmentation of the optical disc and cupping. It can be divided into methods based on image similarity, manual techniques, transfer learning methods, and OD limitation. 

DL methods produce glaucoma diagnosis through image similarity. They need a large dataset for learning networks. Historical and clinical data extracted from the medical consultation can complement DL model features and give reasons for neglected diagnosis results [22].

Manually handcrafted methods used for excavation/disc segmentation are used for feature extraction techniques and ML classifiers using supervised or unsupervised techniques [9,10,11]. However, they exhibit limited accuracy due to the inability to characterize the onset of disease. 

Transfer learning methods are based on pre-trained architectures with non-medical data. They use weights learned from ImageNet. Russakovsky et al. [23] and Gómez-Valverde et al. [13] applied a set with more than 14,000 images to train these networks, although at the cost of lower performance [8].

Methods with OD and OC restriction restrict the analysis area to the OD affected by glaucoma. Limiting the image of the optic papilla allows a better exploration of its features and results in better learning performance of the automated models than at full size but determines a substantial restriction in the field of view of the networks and hinders their ability to learn alternative features of other regions [24]. However, it determines a substantial restriction in the field of view of networks. Furthermore, it hinders their ability to learn alternative characteristics of other regions, for example, the analysis of areas of atrophy of peripapillary fibers, limiting the early diagnosis of glaucoma. 

#### 3.2.2. Classification of Glaucoma by Disc Segmentation and Cupping

Excavation segmentation from a retinal image is challenging due to the lack of depth view in 2D images but is relevant because it helps assess glaucomatous damage to the optic nerve head (ONH) [10]. Furthermore, the papilla region has several anatomical variabilities that are considered normal. It can be interpreted as a false positive by neural networks, such as oblique insertion of the optic nerve, more prominent papillae, and peripapillary atrophies myopia. Most methods use techniques to locate the optic papilla area and then crop the images around it [25,26,27,28,29]. It avoids false positives in regions containing severe illumination artifacts and improves the analysis of the optic papilla but does not differentiate it from other structures of the eye fundus. The precise delineation of the OD is especially difficult in pathological changes such as peripapillary atrophies or hemorrhages [9,11]. Large vessels in the OD area lack depth information in the CFP [8].

## 4. Fundamentals of Public Databases

In this section, generalities of the public databases used in the retinal study are analyzed, as well some reflections on the choice of data with predefined segmentation and partitions, the use of images (without clinical data) for neural network learning, the importance of the quantity and diversity of images, and the evaluation metrics used in the databases. 

### 4.1. Presentation of Public Databases Used for the Retinal Study

Public image bases on the internet contain image clusters used for ophthalmic studies, standardization, and DL architecture research. The databases integrate information obtained through manual processing of the images by experts, can be analyzed by computer algorithms, and allow the comparison of the performance of different algorithms analyzing the same background image. They include, for example, the detection of diabetic retinopathy and diabetic macular edema in fundus photographs [30], lung segmentation [31], brain [32], cell mitosis [33], and the prediction of myopia development and progression [34]. The photo bases were initially designed for use as a database of reference images for segmenting the OD. However, their use has since been more oriented toward the training and testing of deep learning models [19]. 

The images are obtained under multiple conditions, with different cameras, in several groups of patients, with objectives defined by various experts. Therefore, there is difficulty using data and methodologies between the various bases. Just as railways should have homogeneous gauge sizes to avoid frequent changes in compositions, public databases should follow some rules to improve the interchangeability of data and methodologies required, allowing updates of color photographs, classification parameters of glaucoma/non-glaucoma groups confirmed through clinical data, and subsidiary examinations allowing more excellent reproducibility of results, representation of each patient through color photographs of both eyes (because they are non-overlapping images), and centralization of retinal images in pre-established structures. In the very recent work about the “Retinal Fundus Glaucoma Challenge” (REFUGE) [8], a critical step was taken in this direction, suggesting specific criteria that can be used to compare these methods used to classify glaucoma and segment the disc and cup as the availability of publicly accessible image sets, labeled by various experts, with sufficient data for use in DL. The precise separation between training and test sets, enabling the comparison of results [35], the presence of diversity in the image set by various devices, different ethnicities, is obtained from different illumination and contrast, noise, and other conditions, including preliminary diagnosis based on manual reference segmentations of the disc and the cup and providing results from homogeneous methodologies.

New challenges are currently under development, such as the Artificial Intelligence Challenge for RObust Glaucoma Screening (AIROGS), which is to be held to develop solutions for glaucoma screening from CFPs. Still, the study does not detail the protocols that will be followed [36].

The images from the databases intended for the study of glaucoma are generally focused on different anatomical points of the retina. As exemplified in Table 1, many database images contain segmentations from the sternal rim to the papillae and excavation that are made manually by one or more specialists from color nuances, intensity differences, and anatomical structures (vessels) that serve as the basis of classification of glaucoma/non-glaucoma groups. Others are classified manually based on their visuals without additional clinical information, and some are classified based on clinical data.

As can be seen in Table 1, there are few public sets of fundus images to assess glaucoma through the classification or segmentation of the OD and OC. The ORIGIN base [45] database ceased to be public some time ago. However, the Esperanza database cited by Gómez-Valverde et al. [13] has 113 glaucoma images and 1333 normal papillae images. 

Significant discrepancies in the evaluation protocols were observed between the databases. These differences (summarized in Table 1) are mostly related to two key aspects: (i) the datasets used for training/evaluation and (ii) the evaluation metrics [8]. Table 1 also shows the characteristics of various databases used in ophthalmology. Among the databases available on the internet, only DRIHST-GS1, RIM-ONE DL, and REFUGE contain segmentation of the excavation and OD, training, and testing clusters for use in DL architectures and classification of image groups with and without glaucoma. It also shows that the public databases contain a limited number of data covering only a small variability in clinical conditions. Finally, we note that the databases have few public datasets that simultaneously provide segmentation of the OD, cupping, and clinical diagnoses. 

ONHSD [44] and DRIONS-DB [38] include only OD segmentations, and no glaucoma labels are given. ARIA [37] provides OD segmentation and incorporates vessel segmentation and foveal center annotations. However, the images correspond to normal subjects and patients with diabetic retinopathy and age-related macular degeneration, with no cupping segmentation. DR HAGGIS [40], HRF [42], and LES-AV [43], on the other hand, include reliable diagnostic labels and vessel segmentation, but not OD/cavity segmentation. Moreover, their size is relatively small (39, 45, and 22 images, respectively). RIGA [46] is a recent dataset containing 750 fundus images with OD/OC segmentations but no glaucoma classification. As we will see below, RIM-ONE DL includes CFPs from versions v1, v2, and v3 cut around the ONH and includes only OD segmentations made by two specialists [19]. Finally, only DRISHTI-GS [39] and ORIGA [45] include glaucoma classification sets and OD/OC segmentations. However, the diagnostic labels in DRISHTI-GS were assigned solely based on images [39], and DRISHT-GS1 considers a feature of the glaucomatous papilla, the presence of the notch in the neural layer or “notch”. ORIGA, on the other hand, is no longer publicly available.

### 4.2. General Considerations with the Use of Public Databases

The reason to choose databases is based on predefined segmentation and partitions. As seen above, the automated classification of glaucoma directly by DL architectures and through image similarity or pre-trained architectures requires a large dataset for training the networks with great computational expense, representing the major bottleneck of automated diagnosis by DL architectures. In addition, the lack of predefined partitions in training and test sets induces a chaotic practical application of existing data. It affects the direct comparison of the performance of existing methods [35], making it difficult to conclude which features are most appropriate to solve each task.

Surprisingly, there is no information about the source used for diagnostic classification in most existing databases, as indicated in Table 1. Therefore, using images with segmentations but without retrospective analysis of clinical records can be problematic as it may cause bias in automated methods and reproduce mislabeling practices [8]. On the contrary, clinical labels can help algorithms learn and discover other supplementary manifestations of the disease that are still unknown or too difficult to distinguish with the naked eye. Besides that, clinical data can be a diagnostic aid (in suspicious and early cases) and bring a solution closer to the real thing.

Choosing the quantity and diversity of images is important. Existing databases rarely include images obtained from different acquisition devices, ethnicities, or presentations challenging glaucoma-related scenarios, which can interfere with the training of DL networks and result in poor generalization ability. Attempts to address this problem using combinations of different datasets were proposed by Cerentinia et al. [47] and Pal, Moorthy, and Shahina [48], but the results could deviate and influence subsequent evaluations. Incorporating depth information, for example, through stereo imaging and OCT results that would provide dimensions of the excavation and its depth, would ensure reliable annotations. On the other hand, providing segmentations obtained by the consensus of several experts, as in the case of the REFUGE challenge, could better approximate the accurate anatomy by reducing interobserver variability [8]. 

### 4.3. Evaluation Metrics

There is no uniform criterion for comparing methods and evaluating the use of metrics for assessing DL methodologies. Receiver-operating characteristic (ROC) curves are the most commonly used metrics, including the area under the curve (AUC) [13,24,26,34,48]. Sensitivity and specificity [13,24,26,47,49,50] are also used in different studies to complement AUC in driving binary classification results. The authors have reported accuracy [47,51] as another evaluation metric, although this metric may be biased if the proportion of non-glaucomatous images is significantly higher than glaucomatous [52].

## 5. Methodology

The gold standard databases RIM-ONE DL, DRISHTI-GS1, and REFUGE will be analyzed (considered of great interest to applicability in DL architectures) in this section from the point of view of the presentation and characteristics of datasets and results obtained in each base.

The methodology used will compare the databases with the gold standard: RIM-ONE DL [19], DRIHST-GS1 [39,53], and REFUGE [8]. Table 2 summarizes some presentation characteristics of these databases, emphasizing the representation of the segmentation of the disc and optic excavation and the binary classification between glaucoma/non-glaucoma performed by experts and divided into training and test groups. The comparison will be performed based on the presentation and characteristics and the results obtained in each database.

### 5.1. Presentation and Characteristics of the Datasets

#### 5.1.1. RIM-ONE DL

The unified retinal image database for assessing glaucoma using deep learning-RIM-ONE DL [19] is available at https://github.com/miag-ull/rim-one-dl (accessed on 15 June 2022) and was created in 2020 to optimize the three previous versions for use in deep learning. Based on the three previously published versions, a version called RIM-ONE DL (RIM-ONE for Deep Learning) was created and optimized for a deep learning context. All images were again manually segmented (disc and excavation). One image of each eye per patient was kept, and all images were cropped around the optic nerve head using the same proportionality. According to clinical criteria, the images were reclassified into glaucoma and non-glaucoma [8].

The RIM-ONE DL base is divided into two large groups partioned_by_hospital and partitioned_randomly, into test_set and training_set groups. Each group presents subgroups of glaucoma and normal datasets. Table 3 shows the quantitative partitioning of the RIM-ONE DL database datasets.

The images demonstrate the magnification of the region of interest of various features of the glaucomatous papilla. The fundus photographs in the test and training groups present irregularity in sizes, colors, and clarity. It allows only limited analysis of the peripapillary region and hinders the investigation of areas of sectorial atrophy of peripapillary fibers, which is essential for early diagnosis. Moreover, the RIM-ONE DL database presents images randomly obtained between the right and left eye. The classification of normal and glaucoma groups in both test and training sets is based only on segmentations performed manually by experts, no clinical data or ancillary tests are mentioned, and symmetrical images of the eyes are not presented.

#### 5.1.2. DRISHTI-GS1

The Retinal Image Dataset for the Assessment of Glaucoma from the Optic Nerve Head Analysis-DRISHT-GS1 presents a set of retinal images for papillae evolution in normal and glaucomatous eyes with manual segmentation by experts that allows measurements of the ratio of the diameter and cupping and the area of the OD, known as the cup-to-disc ratio (CDR). It presents notch analysis in the superior, inferior, nasal, and temporal (notch) sectors. The author warns about the difficulty of comparing the performance of individual methods due to the lack of a more comprehensive set of bases [39,53]. 

The features presented in Table 2, besides the division into training and test groups, present values of the disc and cupping boundaries and CDR values, i.e., the ratio of cupping/disc measurements. Four experts and an additional expert performed classification into datasets with and without glaucoma based on the segmentation and the extent and direction of notching (I/S/N/T/Absent). 

The measures used for the quantitative analysis of the segmentation method were the segmentation region, boundary location, and the estimated CDR measure. The Hough circle was proposed as the algorithm for the initial segmentation of the optical disc. The DRISHT-GS1 base includes segmentation performed by two more specialists. It is based on three strategies: the detection of the color gradient and the transition of small vessels, the presupposition of the excavation depth by visualizing multiple images, and the presupposition of the excavation depth by color variation (available in the test group) and in-depth maps visualized by OCT (available in the training groups). The author warns of the difficulty in comparing the performance of individual methods due to the lack of a more comprehensive set of bases for classification based on clinical data and divided between the test and training groups [39,53]. The DRISHTI-GS1 database provides a clear division between training/test sets and studies a “notch” feature. Still, it uses a glaucoma classification based only on image features without considering clinical data and ancillary tests.

#### 5.1.3. REFUGE

The Retinal Fundus Glaucoma Challenge (REFUGE) base is available on the website https://refuge.grand-challenge.org/ (accessed on 15 June 2022). It is a competition held as part of the Ophthalmic Medical Image Analysis workshop presented at MICCAI 2018. It was the first initiative to evaluate automated methods for OD/OC segmentation and glaucoma classification from CFPs. For this purpose, the challenge provided the community with 1200 fundus photographs. 

Each image in the REFUGE dataset includes binary glaucoma/non-glaucoma classification performed based on a comprehensive evaluation of patient records, including follow-up fundus images, IOP measurements, OCT images, and visual field. Glaucomatous cases correspond to individuals with glaucomatous damage in the ONH area and reproducible glaucomatous defects. However, early cases or those with pre-perimetric changes and bilateral representation of the right and left papillae are not explained. The images are centered in the posterior pole, between the macula and the visible OD, to evaluate the ONH and potential retinal nerve fiber layer defects.

The REFUGE base presentation is divided into three fixed subsets: training, offline, and online test sets containing equal glaucomatous (10%) and non-glaucomatous (90%) cases. Since training DL from scratch on a training set with only 400 images may lead to overfitting, most groups started the convolutional networks with pre-trained ImageNet weights and then adjusted using the CFPs [8].

**Figure 3:**
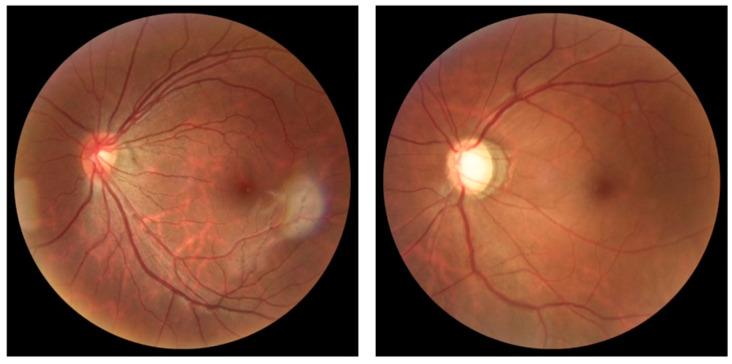
The images T0002.jpg (**left**) with normal excavation and T0010.jpg (**right**) with enlarged excavation were obtained from the test group at 25% of the original size.

**Figure 4:**
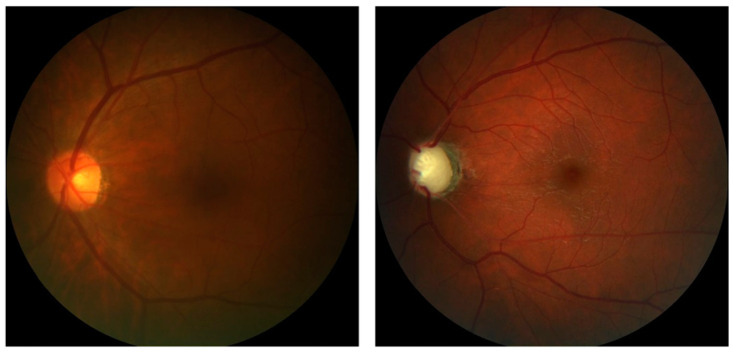
Photos n0002.jpg of the group without glaucoma (**left**) and g0002.jpg of the group with glaucoma (**right**), both reduced to 25% of the original size.

**Figure 5:**
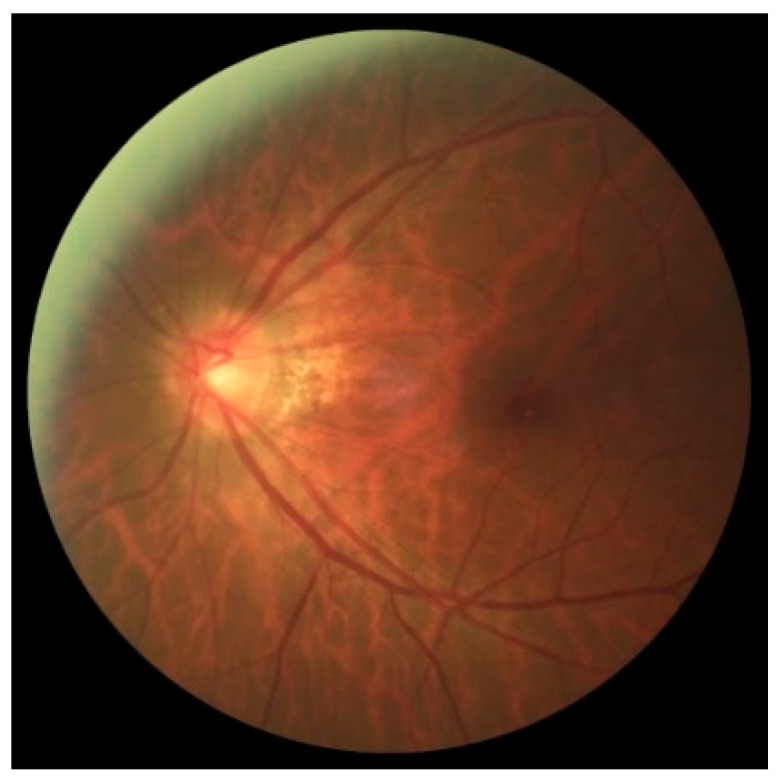
Photo V0002.jpg reduced to 25% of the original size.

The REFUGE base is divided into test (Figure 3), training (Figure 4), and validation (Figure 5) sets. As shown in Table 2, the test set comprises four hundred images of the left eye centered in the papillary macular region. It includes the segmentation performed by a specialist and classification based on clinical data and divided between training and test groups. The training set is divided between 40 photos of the left eye with glaucoma and 160 without glaucoma centered on the papillary macular bundle. The validation set contains four hundred color photographs of the left eye, focusing on the papillary macular bundle. 

Automated methods are currently being developed to predict depth maps of CFPs by correlating ground truth segmentation image features with classifications obtained through other modalities such as stereoscopic photographs. Research is underway in developing automated methods to predict depth maps of CFPs by attempting to correlate image features with ground truth labels obtained from other imaging modalities such as stereo fundus photography [54] or OCT [55]. These techniques may help more reliable glaucoma/non-glaucoma binary classification proposals in databases.

### 5.2. Results of the Databases

According to [19], the RIM-ONE base was widely used in segmentation tasks. However, since 2019 there has been a significant increase in the number of publications associated with deep learning problems, thus reinforcing the need to have a revised and updated version of RIM-ONE to satisfy this new trend. As for the DRISHT-GS1 and REFUGE bases, there is still no information about their use in publications. 

We note that the best approaches for glaucoma classification integrate deep learning techniques with well-known glaucoma-specific biomarkers such as vertical cup changes, cup-to-disc ratio, and retinal fiber layer defects. In the REFUGE challenge, the two top-ranked teams achieved better results than two glaucoma specialists, a promising sign for using automated methods to identify glaucoma suspects with fundus images [8]. U-shaped networks inspired by U-Net [56] were the prevalent solutions. Most teams initialized the CNNs with pre-trained ImageNet weights and then adjusted them using CFPs, because training a DL model from scratch with few data could lead to overfitting, where a model learned patterns specific to the training data and does not generalize well on new, unseen data [8]. Another highlight was that the top-ranked methods in the REFUGE challenge obtained consistently better segmentation results in the subset of glaucomatous individuals than in the non-glaucomatous cases. The most significant excavations (in more advanced cases) present more precise interfaces between the disc and the optical excavation.

#### 5.2.1. RIM-ONE DL

**Figure 6:**
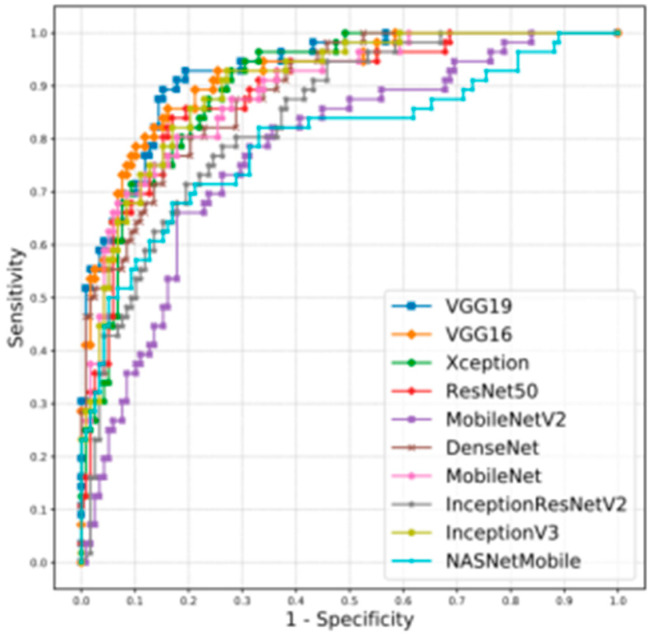
ROC curves for all networks using the Madrid and Zaragoza test set.

The network models VGG19 and VGG 16 had satisfactory results (Table 2). However, a direct comparison with the DRISHT-GS1 and REFUGE challenges is not possible because they present distinctive characteristics of the datasets obtained. Interestingly, the winning team of the REFUGE challenge [57] achieved an AUC of 0.9885 with a sensitivity of 0.9752 (Table 4) for a test sample composed of 360 images from healthy individuals and 40 images from glaucoma patients. Furthermore, in the representation of the ROC curves (Figure 6), is possible to observe a more consistent behavior in the neural networks models using the Madrid and Zaragoza test sets (Figure 6), evaluated in Table 5, whose images were quite different from the images used during training [19].

#### 5.2.2. DRISHTI-GS1

The results were based on the segmentation of the optical disc/scan, the location of the boundary performed by the computed region compared to the ground truth, and the estimation of the CDR and the notch of the inner edge of the neural layer (gap). The initial boundary estimation for optical disc segmentation was based on a transformation algorithm by the Hough circular. Notch segmentation was conducted based on three methods according to the path of the vessels (thin) using a monochrome image, a motion structure to demarcate depth/discontinuity, with false-positive results occurring in the regions of most excellent whiteness, and the segmentation method based on color variation and the corresponding depth of the OCT. The proposed method for notch detection was based on the computational evaluation of neural layer thickness changes against an expert opinion. The edge thickness distribution in normal papillae follows the ISNT rule, which states that the edge thickness decreases along with the sectors of the neural layer in the order Inferior > Superior > Nasal > Temporal, as represented in Table 6, Table 7, Table 8, Table 9 and Table 10.

The ROC curve for the training and testing data is presented below, and the curve values are shown in Table 8. We found a good correlation between notch detection and segmentation achieved through the algorithms by the experts [39].

#### 5.2.3. REFUGE

**Figure 7:**
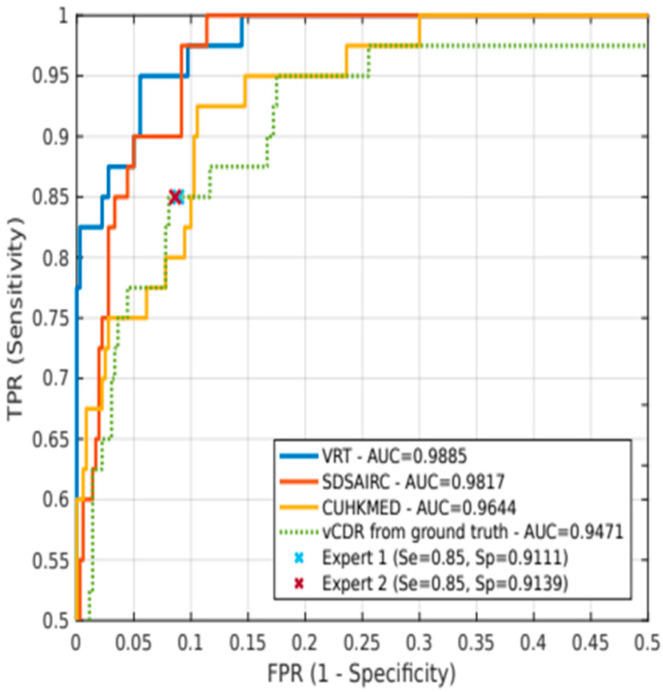
ROC curves and AUC values corresponding to the three top-rated glaucoma classification methods (solid lines) and the vertical cup-to-disc ratio (green dotted line). Crosses indicate the operating points of two experts.

Table 11 shows the results of the participating teams in the REFUGE test set. The VRT team (winning team of the challenge) achieved an AUC of 0.9885 and a vertical cup-to-disc ratio (vCDR) of 0.9752. The last row corresponds to the results obtained using the ground truth vCDR. Two glaucoma experts who were not part of the group of experts that provided the ground truth segmentations visually classified the images from the test set and assigned a binary glaucomatous/non-glaucomatous label to each. A different approach based on ground truth vCDR values was also included as a probability for glaucoma classification. The VRT team had the best classification performance, achieving significantly better results than vCDR. The evaluation of the classification task, in terms of AUC and the reference sensitivity with 85% specificity, is presented in Figure 7. The AUC measures the quality of the model predictions, regardless of the classification threshold. Despite referring to glaucoma/non-glaucoma classification, the databases used different parameters, making it difficult to compare the results between the databases. 

REFUGE contains OD/OC masks, fovea positions, and gold-standard clinical diagnostic classification. This feature aids glaucoma classification methods as it was recently observed that training with categories made by CFPs harms performance in detecting truly diseased cases [58].

## 6. Discussion

Analysis of the results and recommendations will be made to improve the databases and make cross-sectional annotations in the dataset feasible. The public databases’ main limitations and the results’ clinical implications are discussed below.

The bases RIM-ONE-DL [19], DRISHTI-GS [53], and REFUGE [8] have proved to be beneficial for the automated study of the papilla by including the following features: classification between normal and glaucoma eyes, segmentation of the OD, and excavation and differentiation of the test and training groups. The DRISHTI-GS database contains images with the centralized optic papilla and a periphery of about 30 degrees around the papilla. It allows the visualization of the excavation features and sectorial defects in the fiber layers, an element considered in early classification. The REFUGE database includes groups of normal and glaucoma eyes pre-classified based on the evaluation of the patient’s clinical records (not in the presentation), including fundus images, ocular pressure measurements, OCT images, and visual field, with images of glaucoma eyes corresponding to patients with reproducible glaucomatous damage. Only REFUGE and RIM-ONE DL meet the additional requirements of offering images from different cameras and clear training and test data [8]. Only the REFUGE database was classified based on clinical records or subsidiary examinations.

A synopsis of the main advantages and disadvantages of the gold standard public databases used in glaucoma is shown in Table 12.

As analyzed in Trucco et al. [35], the lack of predefined partitions in training and test sets induces a chaotic practical application of existing data that affects the direct comparison of the performance of existing methods, making it difficult to conclude which features are more appropriate to solve each task. 

### 6.1. Recommendations to Improve the Databases

The recommendations for improving the databases suggest a set of protocols to avoid errors in training and make the training of DL nets closer to reality.

1. Data should be continually updated to encompass the normal and pathological anatomical diversities of the fundus of the eye.
2. There should be a more representative dataset including a diversity of ethnicities, comorbidities, genders, and ages; comparative photographs of both eyes; and color fundus photographs with varying qualities.
3. A percentage of glaucoma cases should be maintained that is similar to that expected in a population-screening scenario estimated to be between 1% and 2% in the general population, reaching 6% to 7% after 70 years [59].
4. Manual disc and cupping segmentations and binary ground truth classifications performed from CFPs should consider cupping depth information from OCT scans, which provide cross-sectional retinal images (and thus depth information) and reference Bruch’s membrane, which is considered to be the best anatomical delimitation of the OD, and serves as a reference for one of the most recent measurements of the number of retinal nerve fibers (BMO-MRW) [60]. The complementarity of CFP and OCT for automated glaucoma screening still needs to be explored.
5. Protocols should be made available for the analysis of medical records used as the basis for glaucoma/non-glaucoma binary classification to allow other comorbidities that may accompany glaucoma to be addressed.

### 6.2. How Public Databases Can Contribute

The databases can contribute to more reliable and accurate results for the training/testing groups, increasing the accuracy of the screening results of the DL networks regarding the segmentation and binary classification of the normal/glaucomatous papilla through images.

### 6.3. Feasibility of Annotating across All Datasets

Cross-sectional annotation of datasets using homogeneous protocols could increase the available data and improve the network results. Therefore, to validate a cross-sectional annotation, the images should be repositioned and submitted to homogeneous criteria of disc segmentation and cupping, and a re-analysis of the binary classification between normal/glaucomatous papillae based on clinical data and ancillary tests such as visual field and OCT should be performed, since the analysis by image similarity increases the number of false positives, as reported. The grouping of images with irregularities not yet visible at the inner border of the neural layer but with corresponding initial perimetric alterations would bring advantages in the early diagnosis. Sets of betterand worse-quality images obtained by different cameras in distinct locations and populations could be used in the training of neural networks as parameters closer to reality. Another point of divergence for the network is the presentation of the right papilla image and how the networks would interpret the sample of the left papilla since the papillae of the two eyes do not overlap spatially. Another factor is that the databases cannot be matched as they use different strategies. Protocols with an approach closer to the real world would provide greater sampling security. 

### 6.4. Notch Detection

No single parameter would bring a diagnostic confirmation of glaucoma. Some parameters such as ocular hypertension and glaucomatous papilla features, including enlarged cupping, are factors for suspecting the disease. The diagnostic certainty of glaucoma consists in the correspondence between the glaucomatous part of the papilla with an alteration in the visual field in that region. The same reasoning can be applied to the presence of a notch. This is a feature that can be considered suspicious. Still, it is not pathognomonic of glaucoma by itself, as it may be only an image noise that does not represent a region of neural damage. The detection of a (small) notch correlated with the visual field may be a goal to be pursued by AI as far as it facilitates early diagnosis.

### 6.5. Limitations of Public Databases

1. Retinal image pigmentation can undergo a few changes in different ethnicities and influence the performance of others.
2. The percentage of glaucoma cases in the REFUGE, DRIHST-GS1, and RIM-ONE datasets is higher than expected in a screening setting.
3. Including only high-quality photographs makes applying the proposed methods in real screening scenarios challenging.
4. Manual OD/OC segmentations performed from CFPs may be ill-defined.
5. Better binary ground truth classifications follow two-dimensional patterns for delineating OD/OC.
6. Color photographs do not match data from analyzed medical records and do not allow a broader approach to other comorbidities that may accompany glaucoma.

### 6.6. Clinical Implications and Future of Databases

Reliable results obtained within a dataset presented in one database may not necessarily reflect the same results in other databases subjected to the same learning values considering heterogeneous protocols (as cited). The automated systems can detect suspected glaucoma cases from fundus photographs as long as they follow some previously analyzed protocols. At the moment, this is still an open question. Although challenges such as REFUGE are moving in a more realistic direction, we do not yet know the significance of the answers that AI could provide in a population setting. With technological development continuously transforming, fundus cameras are improving portability and ease of use, as well as the use of defined protocols. It is hoped to expand the use of AI to diagnose and follow the progression of glaucomatous disease, remembering that the modality of early imaging in databases is still pending due to the subtle manifestation of the initial stages of disease images. 

We saw that some of the proposed models were able to identify glaucoma with a focus on a few biometric features of the optic papilla, such as the disc-to-cavity ratio in REFUGE and the notch in DRISHT-GS1. We have seen that the REFUGE challenge results also seem to indicate that the use of vCDR may be a feature of greater importance than others, such as ONH hemorrhages, sectorial atrophies, or RNFL defects. Based on vCDR, as a probability of glaucoma, sensitivity and specificity values were statistically equivalent to those obtained using ground truth. Perhaps these parameters alone do not diagnose the disease but may have importance as single screening factors. However, we may incur errors by basing the binary classification on only a few features. Therefore, it is essential to have a conveniently classified sample of data.

In the REFUGE challenge, the top-performing teams complemented ONH measurements by DL models. We can significantly outperform expert diagnosis of glaucoma, with increases in sensitivity of up to 10% [8]. Although these results are limited to a specific population, we can still argue that these deep learning models can identify complementary features that are invisible to the naked eye and are essential to ensure a more accurate disease diagnosis. 

Other metrics derived from relative OD/OC shapes have recently outperformed vCDR in the diagnostic process, such as the rim-to-disc ratio [61]. However, some clinical guidelines, such as the European Glaucoma Society 2017 [62], do not recommend vCDR to classify patients, as several healthy discs may have large vCDR. Instead, the focus is on neuroretinal rim thickness (ISNT rule) and the degree of vCDR symmetry between eyes. In any case, vCDR is still a relevant parameter (it reached an AUC of 0.9471 in the test set for glaucoma classification). In addition, other ophthalmological parameters such as ocular pressure; the asymmetry between the papillae; the presence, size, and location of ONH hemorrhages; or the presence and size of retinal fiber layer defects can help analyze disease progression in each patient visit to ensure more reliable predictions. 

Retinal evaluation by CFPs allows for the cost-effective assessment of glaucoma. Although OCT better highlights the excavation and fiber layer damage and provides a three-dimensional view of the retina, its large-scale use is economically unfeasible. Other markers used alone to make transverse scans and quantify the thickness of the RNFL or the size of the excavation have proven to be financially unviable and alone do not confirm the diagnosis. Thus, the development of DL methods for glaucoma screening must integrate CFPs analysis and glaucoma biomarkers.

Therefore, we recommend a database with more robust data following some protocols that allow the integration between datasets that may encompass CFPs obtained from multi-population centers, with the segmentation of the OD and cupping and images of both eyes, with diversified image quality, divided into well-defined training/test/validation groups, presenting structured binary classifications using clinical criteria that allow division into groups of papillae with early to severe glaucoma, besides the possibility of analyzing other retinal comorbidities evaluation through methods.

## 7. Conclusions

Public databases form the training and learning base for automated screening performed by deep learning architectures. Of the various databases used in ophthalmology, many are most helpful in diagnosing diabetic retinopathy, senile macular degeneration, and glaucoma. Among the bases used for glaucoma, the gold standards RIM-ONE DL, DRISHTI-GS1, and REFUGE are the most useful because they present segmentations of the disc and the excavation drawn by specialists and present classifications of normal papillae and those with glaucoma based on images, clinical data, and ancillary tests. Its disadvantages are the heterogeneous photographic quality, varied sizes and positioning of the image, and lack of images of both eyes. It would be advantageous to develop a protocol capable of performing population screening for glaucoma and diagnosing early forms of the disease and other retinal comorbidities in the medical context. Therefore, we suggest that the database data be continually updated and reclassified based on image segmentation and using clinical data and ancillary tests to provide more robust data to public databases and make the results more dependable. 

Deep learning architectures could be made more dependable by using other clinical biomarkers, symptoms, eye pressure, family, and personal history to make DL models closer to the real thing and to make the results more accurate. 

### Future Work

We suggest the inclusion of clinical parameters such as notch (neural portion defects), CDR measurements as performed in the DRISHT-GS1 database, images with pre-perimetric defects, images with non-glaucomatous peri-papillary changes (myopic degeneration, peri-papillary atrophy), and continual updating of images to encompass rare diagnoses and avoid false positives. 

The possibility of including the simultaneous screening of other retinal pathologies concomitant to glaucoma in DL networks in clinical practice should be screened by specialists through clinical data or be diagnosed as an examination finding. In this case, color retinography is significant in diagnostic aid and will certainly demand an excellent function of the public databases and algorithms to embrace a more comprehensive diagnostic range.

## Figures and Tables

**Table 1:** Comparison of the dataset with other publicly available databases of background images. Question marks indicate missing information, and N/A means “not applicable”. Adapted from [8].

Dataset	Num. of Images			Ground Truth labels			Different Cameras	Training/ Test Split	Diagnosis from	Evaluation Framework
	g+	g-	Total	Class of Glaucoma	Segmentation Disc/ Excavation	Location of the Fovea?				
**ARIA** [37]	0	143	143	on	yes/no	yes	on	on	?	on
**DRIONS-DB** [38]	-	-	110	on	yes/no	on	?	on	N/A	on
**DRISHTI-GS1** [39]	70	31	101	yes	yes/no	on	on	yes	images	on
**DR HAGGIS** [40]	10	29	39	yes	no/no	on	yes	on	clinical	on
**Madrid** [41]	0	516	516	on	yes/no	yes	on	yes	?	yes
**HRF** [42]	15	30	45	yes	no/no	on	on	on	clinical	on
**SLE-AV** [43]	11	11	22	yes	no/no	on	on	on	clinical	on
**ONHSD** [44]	-	-	99	on	yes/no	on	on	on	N/A	on
**ORIGIN** [45]	168	482	650	yes	yes/no	on	?	on	?	on
**RIM-ONE DL** [19]	172	313	485	yes	yes/no	on	yes	yes	images	on
**RIGA** [46]	-	-	750	on	yes/no	on	yes	on	?	on
**REFUGE** [8]	120	1080	1200	yes	yes/no	yes	yes	yes	clinical	yes

g+ glaucoma, g- normal, N/A “not applicable,” (?) missing information.

**Table 2:** Summarizes the characteristics of gold standard public databases.

	Format	Normal and Glaucoma Eyes	Training Group	Test Group	Segmentation		Diagnostic Elements	Simultaneous Imaging OD/OE
					Right	Left		
**RIM ONE-DL**	.png	313-/172+	195-/116+	118-/56+	(+)	(+)	clinical	not
DRISHTI-GS1 *	.png	31-/70+	50	51	(+)	(+)	image	not
**REFUGE**	.jpeg	1080-/120+	360-/40+	400 offline400 online	(+)	(+)	clinical	not

* Presence or absence of the notch is analyzed on a DRISHTI-GS1 basis. Normal eyes (-) with glaucoma (+) optic disc (OD), cupping (ESC), segmentation present (+), and absent (-).

**Table 3:** Presentation of the RIM-ONE DL database datasets.

	TEST_SET		TRAINING_SET	
	Normal	Glaucoma	Normal	Glaucoma
**PARTIONED_BY_HOSPITAL**	118	56	195	116
**PARTITIONED_RANDOMLY**	52	94	219	120

**Table 4:** Evaluation of different networks using the randomized test set.

Network	AUC	Se	Acc.
**VGG19**	0.9867	1.0000	0.9315
**VGG16**	0.9834	0.9615	0.9247
**Xception**	0.8771	0.9808	0.9178
**ResNet50**	0.9755	0.9808	0.9110
**MobileNetV2**	0.9738	0.9423	0.9041
**DenseNet**	0.9726	0.9615	0.9041
**MobileNet**	0.9712	0.9615	0.9315
**InceptionResNetV2**	0.9685	0.9808	0.9110
**InceptionV3**	0.9597	0.9423	0.8904
**NASNetMobile**	0.9290	0.9231	0.7534

**Table 5:** Evaluation of networks using the Madrid and Zaragoza test suite.

Network	AUC	Se	Acc.
**VGG19**	0.9272	0.8750	0.8563
**VGG16**	0.9177	0.8214	0.8506
**InceptionV3**	0.9015	0.7500	0.8046
**Xception**	0.8982	0.7500	0.7989
**DenseNet**	0.8919	0.7143	0.7816
**MobileNet**	0.8912	0.7500	0.8276
**ResNet50**	0.8855	0.7321	0.8333
**InceptionResNetV2**	0.8396	0.625	0.7644
**NASNetMobile**	0.7969	0.6071	0.7989
**MobileNetV2**	0.7765	0.4464	0.5287

**Table 6:** Distribution of normal/glaucomatous eye images and notch cases in the training and test sets—adapted from [39].

Notching		Diagnosis		
Absent	Present	Glaucomatous	Normal	
31	19	32	18	Train
25	26	38	13	Test

**Table 7:** Optical disc segmentation results—adapted from [39].

Test		Train	
Boundary Localization Error (Pixels)	F-Score	Boundary Localization Error (Pixels)	F-Score
8.93 ± 2.96	0.96 ± 0.02	8.61 ± 8.89	0.96 ± 0.05

The table entries represent means ± standard deviation obtained in images.

**Table 8:** Excavation segmentation results—adapted from [39].

Test		Train	
Boundary Localization Error (Pixels)	F-Score	Boundary Localization Error (Pixels)	F-Score
30.51 ± 24.80	0.77 ± 0.20	33.91 ± 25.14	0.74 ± 0.20
25.28 ± 18.00	0.79 ± 0.18	24.24 ± 16.90	0.77 ± 0.17
21.21 ± 15.09	0.81 ± 0.16	22.10 ± 19.47	0.80 ± 0.18

The table entries represent means ± standard deviation obtained in images.

**Table 9:** Error in CDR estimation in OD segmentation and excavation (deviation ± mean) evaluated against different experts—adapted from [39].

Test	Train	
0.18 ± 0.14	0.15 ± 0.12	Expert 1
0.17 ± 0.11	0.13 ± 0.10	Expert 2
0.13 ± 0.12	0.10 ± 0.10	Expert 3
0.14 ± 0.12	0.11 ± 0.11	Expert 4
0.16 ± 0.02	0.12 ± 0.02	Average

**Table 10:** Performance of the proposed method for notch detection—adapted from [39].

	**Area Under Curve**	**Sensitivity**	**Specificity**
**Training Set**	0.81	0.84	0.71
**Test Set**	0.79	0.81	0.72

**Table 11:** Ranking results of the participating teams in the REFUGE test set. The last row corresponds to the results obtained using the vCDR.

Rank	Team	AUC	Reference Sensitivity
**1**	**VRT**	**0.9885**	0.9752
2	SDSAIRC	0.9817	**0.9760**
3	CUHKMED	0.9644	0.9500
4	NKSG	0.9587	0.8917
5	Mammoth	0.9555	0.8918
6	Masker	0.9524	0.8500
7	SMILEDeepDR	0.9508	0.8750
8	BUCT	0.9348	0.8500
9	WinterFell	0.9327	0.9250
10	NightOwl	0.9101	0.9000
11	Cvblab	0.8806	0.7318
12	AIML	0.8458	0.7250
Ground truth vCDR		0.9471	0.8750

**Table 12:** Main advantages and disadvantages of gold standard databases.

Databases	Advantages	Disadvantages
**RIM-ONE DL**	- Segmentation by five specialists - Classified from clinical data - Division into test/training groups	- It does not show symmetrical images between the two eyes. - Image is cut around the optical disc. - There are no clinical data or examinations corresponding to each dataset.
**DRISHTI-GS1**	- Segmentation by five specialists - Classification based on clinical notch findings, CDR, and examinations - -Divided between training and test group	- It does not show symmetrical images between the two eyes. - There is a small number of data and experts. - Glaucoma/non-glaucoma classification is conducted based on image feature analysis. - There are no clinical data and examinations corresponding to each dataset.
**REFUGE**	- A larger number of images - Includes segmentation by experts - Classification based on clinical data - Divided between training and test group	- Sampling is limited to a specific population. - It does not show symmetrical images between the two eyes. - The image is not centered on the papilla. - There is no access to the patient’s clinical data with prejudice to the access of other comorbidities.

## Data Availability

Not applicable.
